# Long-term probability of intraocular pressure elevation with the intravitreal dexamethasone implant in the real-world

**DOI:** 10.1371/journal.pone.0209997

**Published:** 2019-01-04

**Authors:** Javier Zarranz-Ventura, Anna Sala-Puigdollers, Daniel Velazquez-Villoria, Marc Figueras-Roca, Sergio Copete, Laura Distefano, Anna Boixadera, Jose García-Arumi, Alfredo Adan, on behalf of the Hospital Clínic—Hospital Vall de Hebron Intravitreal Dexamethasone Implant study group

**Affiliations:** 1 Institut Clínic d´Oftalmología (ICOF), Hospital Clinic, Barcelona, Spain; 2 Departmento de Oftalmología, Hospital Vall de Hebron, Barcelona, Spain; Massachusetts Eye & Ear Infirmary, Harvard Medical School, UNITED STATES

## Abstract

**Purpose:**

To evaluate the long-term cumulative probability of intraocular pressure (IOP) elevation with the intravitreal dexamethasone implant (IDI) when used to treat different indications: diabetic macular edema, uveitis, retinal vein occlusion.

**Methods:**

705 IDI injections (429 eyes) were assessed and Kaplan-Meier graphs were generated to assess: the probability of different levels of IOP elevation (IOP≥21, ≥25 or ≥35 mmHg), IOP change ≥10 mmHg, initiation of IOP-lowering treatment, glaucoma surgery, IOP change with repeat injections and IOP elevation in eyes with glaucoma and ocular hypertension (OHT).

**Results:**

The cumulative probability of IOP ≥21, ≥25 and ≥35 mmHg was 50%-60%, 25%-30% and 6%-7% at 12–24 months, respectively. The probability of initiating IOP-lowering medication was 31%-54% at 12–24 months. Glaucoma and OHT eyes had a higher probability of mild IOP elevation (≥21 mmHg, 65.1%, 75% and 57.8%, p = 0.01), yet a similar moderate (≥25 mmHg, 22.3%, 28% and 30.2%, p = 0.91) and severe elevation of IOP (≥35 mmHg, 3.7%, 7.1% and 4%, p = 0.71) as normal eyes. Glaucoma surgery was required in only 0.9% cases (4/429). At baseline, 8.8% of the treated eyes had glaucoma, 6.7% OHT and 16.9% were already on IOP-lowering medication.

**Conclusions:**

In the long-term (24 months), IOP elevation is common, generally mild (30% IOP, ≥25 mmHg) and well-tolerated, resolving with topical treatment (54%) and rarely requiring surgery (0.9%).

## Introduction

The intravitreal dexamethasone implant (IDI: Ozurdex, Allergan, Inc. Irvine, CA) is a bioerodible device composed of polylactic acid and polyglycolic acid polymers that releases 0.7 mg of preservative-free dexamethasone for up to 6 months.[1–4] Its efficacy has been confirmed in clinical trials for the management of a variety of retinal diseases, such as macular edema (ME) related to retinal vein occlusion (RVO), non-infectious uveitis or diabetic macular edema (DME).[1,5–8] However, the applicability of these results to the real world is limited as it is unclear whether these outcomes reflect the use of the implant in routine clinical care given the strict entry criteria and high frequency of follow up visits in clinical trial cohorts. As a result, significant differences have been reported in treatment patterns, reinjection frequency and clinical outcomes for each of the aforementioned indications.[9–14] More importantly, while the safety profile of the implant has been well described in clinical trials, there is a paucity of information in clinical settings, where it is used for a wider range of indications, with less frequent follow up visits and indeed, often including the treatment of patients with ocular hypertension (OHT) or glaucoma.

Elevations in intraocular pressure (IOP) and cataract formation are common and well-documented side effects of intravitreal steroids, and the IDI is no exception.[2,15,16] In the longest follow up clinical trial, the three-year, randomized, sham-controlled dexamethasone intravitreal implant in patients with Diabetic Macular Edema (MEAD) trial, the percentage of eyes with a significant rise in IOP (defined as IOP ≥25 mmHg or IOP change from baseline ≥10 mmHg) was 32% and 27.7%, respectively.[1] In the same cohort, 41% of the eyes required IOP-lowering topical treatment while only 0.6% required glaucoma surgery within the 3 years. Given that the trials for other indications were designed with a shorter follow-up period, ranging from 6 to 12 months, these 36-month data appear to be a reliable long-term estimate of IOP-related problems with the IDI in a clinical trial scenario. In the real world, some recent studies that set out to assess the efficacy of the IDI also provided data about its safety profile outside the controlled clinical trial conditions.[11,15] However, the majority of these studies only report short-term data (6 months) and they have not specifically addressed the cumulative probability of IOP elevation over longer follow-up periods. Due to the difficulty of obtaining reliable long-term data in a clinical setting, there is little information regarding the long-term safety of the IDI in real world practice.

As a result, our aim was to establish a collaborative project between two specialized retinal units in a well-defined geographical area (Barcelona, Spain) to evaluate how the IDI is used in routine clinical care. In particular, this study set out to ascertain the long-term probability of elevations in IOP and related factors in a large cohort of eyes treated with the IDI in the real world.

## Methods

This study was approved by the Institutional Review Board of Hospital Clinic of Barcelona as part of a non-interventional retrospective audit, and it was conducted in accordance with the Tenets set forth in the Helsinki Declaration. Clinical data were collected retrospectively from 2 tertiary referral specialist retina clinics in Barcelona, Spain: Institut Clínic de Oftalmología-ICOF at the Hospital Clinic Barcelona; and the Hospital Vall de Hebrón. During a 55 month period (October 2010 –May 2015), all eyes that received IDI injection for any indication at these units (i.e. DME, RVO, non-infectious uveitis or off-label use) were included in the study. A comprehensive data spreadsheet was used in both centers and it was completed by May 2015. Patient identifiers were removed to make the data anonymous, and the data from the individual centers were collated and merged into a centralized database for analysis.

### Clinical data

The data collected included demographics (i.e. age, gender, etc.), laterality, indication for treatment, number of injections, IOP-lowering topical treatments, previous local treatments, surgical details, complications, and IOP at baseline, 1–2 weeks, 6–8 weeks, 3, 6, 9, 12, 18 and 24 months post-injection of the first IDI. Moreover, for each individual repeat injection during the study period, IOP data was collected prior to the procedure and 1–2 weeks, 6–8 weeks and 3 and 6 months post-injection.

### Data sources/measurements

As the data were gathered from routine clinical settings, IOP was measured in mmHg by Goldmann tonometry at each time point. Analysis for eyes with low VA was undertaken by substituting counting fingers (CF) and hand movement (HM) with 2.0 and 2.3 logMAR, respectively.[17] In patients where data were not available for a particular visit or had been lost to follow-up, no missing value substitution was performed. Descriptive percentages at different time points were estimated as per the existing data for this specific time point.

This study specifically aimed to evaluate the IOP outcomes of the study cohort. The visual and anatomical outcomes of the implant in each specific indication (DME, uveitis, RVO and off-label use) will be reported elsewhere.

### Statistical analysis

The cumulative probabilities of events occurring after IDI injection are presented as survival curves using the Kaplan Meier (K-M) method.[18] Elevated IOP was defined as an IOP ≥21 mmHg. As described elsewhere [16], the cumulative probability of IOP ≥25 mmHg, ≥35 mmHg and an IOP change from baseline was calculated and represented in the K-M survival curves. The probability of IOP elevation to different levels was evaluated for the first, second and third IDI injection. Subgroup analysis was performed on normal, OHT and glaucoma eyes, and K-M survival curves were compared with the log-rank test. Descriptive, frequency statistics and the chi-squared test were used to assess the qualitative variables. Normality of the quantitative variables was examined using histograms. The paired t-test was used to compare pre- and post-treatment changes of the mean when variables were distributed normally, and the Wilcoxon test in cases where non-parametric tests were required. A *p* value of less than 0.05 was considered statistically significant and all statistical analyses were performed using Excel and SPSS 21.0 software (IBM SPSS Statistics v21.0, Armonk, NY, IBM Corp).

## Results

### Baseline characteristics of the study eyes

Data was received for 705 IDI injections performed on 429 eyes from 403 patients over a 55 month period, with a mean follow up of 16.4±8 months (median 17, interquartile range–IQR- 14.3) and 8.4% of patients receiving bilateral injections (34/403). The most frequent indication for treatment was DME (46.3%, 203/429), followed by RVO (28%, 123/429), Uveitis (13%, 56/429) and other off-label indications (10.9%, 47/429). At baseline, 7.9% of the study eyes (32/402) had an IOP ≥21 mmHg, 6.7% were diagnosed with OHT (29/428), 8.8% had glaucoma (38/428) and 16.9% were already on treatment with topical hypotensive drops (72/425). Patient demographics, the number of injections, phakic status, the mean baseline IOP, as well as the percentage of study eyes with OHT, glaucoma and eyes on IOP-lowering medication at the moment of the first injection are detailed in **Table 1**.

**Table 1:** Baseline characteristics of the study eyes, total and relative to the indication: number of eyes, number of injections, mean age, gender distribution, phakic status (percentage of phakic patients), mean IOP, ocular hypertension and glaucoma eyes at baseline, as well as the percentage of eyes on topical hypotensive treatment at baseline.

	Total	RVO	Uveitis	DME	Others
**N** (%)	429 (100%)	123 (29%)	56 (13%)	203 (47%)	47 (11%)
**Number of injections**	705 (100%)	205 (29%)	94 (13%)	342 (49%)	64 (9%)
**Age** (Mean±SD)	65.3 ±13.3	67.4 ±12.2	51.3 ±17.4	66.8 ±10.3	68.5 ±13.2
**Gender** (%F, F:M)	50.3% (203:200)	53.6% (66:57)	60.4% (29:19)	43.8% (78:100)	51.0% (30:24)
**Phakic status** (%P, P:PseudoP)	51.8% (219:203)	72.1% (88:34)	43.3% (23:30)	51.0% (102:98)	12.7% (6:41)
**IOP (mmHg)** (Mean±SD)	15.7 ±3.4	15.6 ±3.0	13.7 ±4.2	16.0 ±3.1	16.8 ±3.6
**OHT** (%)	6.7% (29/428)	2.4% (3/122)	8.9% (5/56)	8.9% (18/202)	6.25% (3/48)
**Glaucoma** (%)	8.8% (38/428)	9.0% (11/122)	8.9% (5/56)	9.4% (19/202)	6.25% (3/48)
**IOP-lowering Treatment** (%)	16.9% (72/425)	12.1% (15/123)	16.9% (9/53)	20.2% (41/202)	14.8% (7/47)

### Probability of elevated intraocular pressure

**Fig 1:**
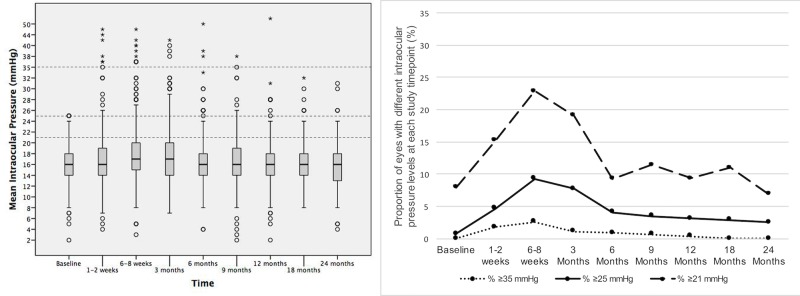
Intraocular pressure (IOP) at each time point after the first IDI injection. Left: Box-plots showing the distribution of the IOP of all eyes at each time point after the first IDI injection. The horizontal lines in each box represent the median for each group, the limits of the boxes are the upper and lower quartiles, with the whiskers representing the minimum and maximum values, and the circles and stars representing the outliers. The dotted lines represent the IOP levels of interest (≥21, ≥25 and ≥35 mmHg). Right: Proportion of eyes with different IOPs at different time points after the first IDI injection.

The mean IOP for all the study eyes, the percentage of eyes with an IOP <21, ≥21, ≥25, ≥35 mmHg, the eyes in which there was a change in IOP ≥10 mmHg and the eyes receiving IOP lowering medication are described at all time points in **Table 2 and Fig 1**.

**Table 2:** Distribution of intraocular pressure outcomes (IOP) during the study period.

	Baseline	1–2 weeks	6–8 weeks	3m	6m	9m	12m	18m	24m
**Mean IOP** Mean±SD	15.7±3.4	16.9±5.1	18.0±5.6	17.5±5.1	16.4±4.5	16.6±4.3	16.2±4.5	16.5±3.8	15.9±4.0
**% Eyes with IOP < 21 mmHg**	92.0 (370/402)	84.8 (328/387)	77.1 (300/389)	80.9 (292/361)	90.7 (312/344)	88.6 (256/289)	90.6 (261/288)	89.0 (186/209)	93.1 (148/159)
**% Eyes with IOP ≥ 21 mmHg**	7.9 (32/402)	15.2 (59/387)	22.9 (89/389)	19.1 (69/361)	9.3 (32/344)	11.4 (33/289)	9.4 (27/288)	11.0 (23/209)	6.9 (11/159)
**% Eyes with IOP ≥ 25 mmHg**	0.7 (3/402)	4.6 (18/387)	9.2 (36/389)	7.7 (28/361)	4.0 (14/344)	3.4 (10/289)	3.1 (9/288)	2.8 (6/209)	2.5 (4/159)
**% Eyes with IOP ≥ 35 mmHg**	0 (0/402)	1.8 (7/387)	2.5 (10/389)	1.1 (4/361)	0.8 (3/344)	0.6 (2/289)	0.3 (1/288)	0 (0/209)	0 (0/159)
**% Eyes with IOP change from baseline ≥ 10 mmHg**	-	2.9 (11/370)	8.3 (31/371)	7.8 (27/343)	4.0 (13/324)	5.4 (15/277)	2.5 (7/275)	1.4 (3/201)	5.7 (9/156)
**% IOP lowering treatment**	16.9 (72/425)	19.3 (80/414)	26.4 (109/412)	22.7 (88/387)	21.6 (83/384)	21.6 (77/356)	21.0 (74/351)	19.7 (64/324)	19.6 (57/290)
***(Total n = 429)***	***(n = 402)***	***(n = 387)***	***(n = 389)***	***(n = 361)***	***(n = 344)***	***(n = 289)***	***(n = 288)***	***(n = 209)***	***(n = 159)***

Mean IOP at different time points. Percentage of eyes with different IOP levels [% at each time point (number of eyes/total number of eyes)]: IOP, intraocular pressure; SD, Standard deviation; 1m, 1 month; 3m, 3 months; 6m, 6 months; 12m, 12 months; 18m, 18 months; 24m, 24 months).

**Fig 2:**
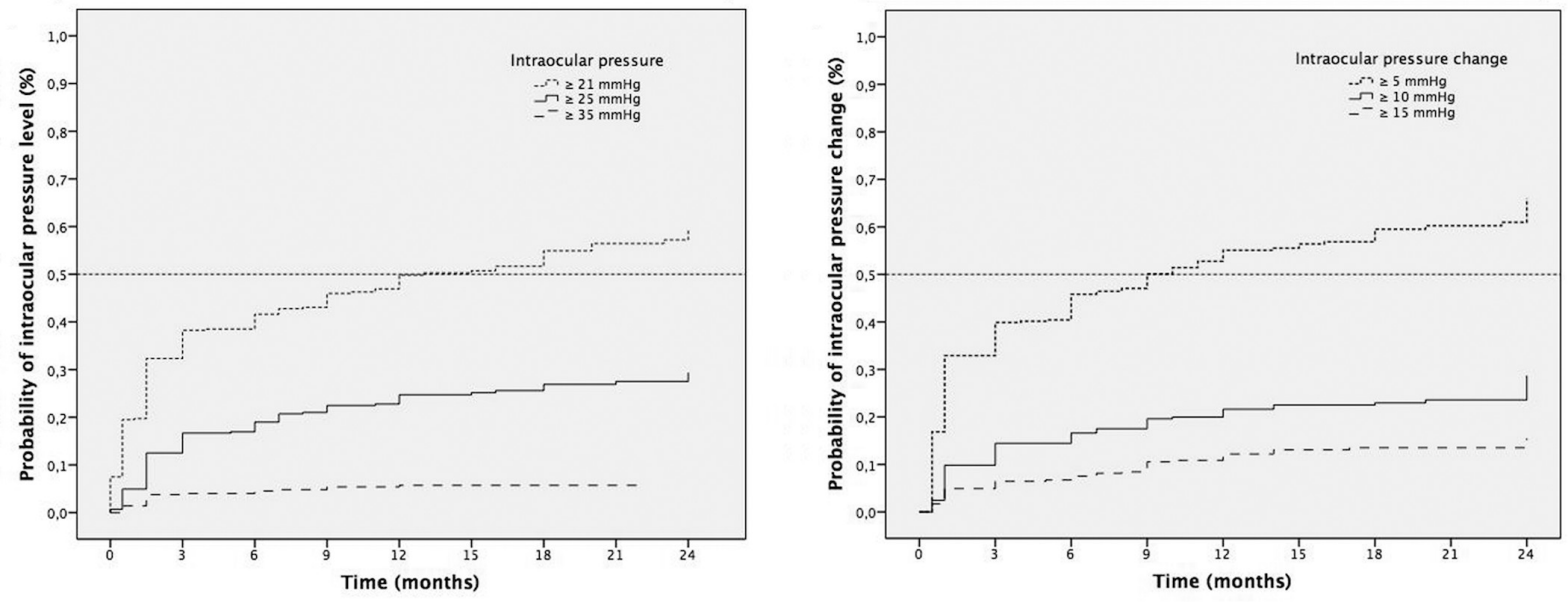
Kaplan-Meier survival analysis of the time to reach an elevated intraocular pressure (IOP) and the change in IOP within 24 months of injection (in months). Left: Cumulative probability of the different increases in IOP in the months after the first IDI injection (dotted line = IOP ≥21 mmHg, solid line = IOP ≥25 mmHg, stripped line = IOP ≥35 mmHg). Right: Cumulative probability of the different changes in IOP relative to the baseline in months (dotted line = IOP change ≥5 mmHg, solid line = IOP change ≥10 mmHg, stripped line = IOP change ≥15 mmHg).

The cumulative probability of having an IOP ≥21 mmHg was 20% at 1–2 weeks, increasing to 33% at 6–8 weeks, 39% at 3 months, 42% at 6 months, 50% at 12 months, 55% at 18 months and 60% at 24 months. The probability of having an IOP ≥25 mmHg was 5% at 1–2 weeks, 13% at 6–8 weeks, 17% at 3 months, 19% at 6 months, 25% at 12 months, 27% at 18 months and 30% at 24 months. Finally, the probability of having an IOP ≥35 mmHg was 2% at 1–2 weeks, 4% at 6–8 weeks, 4% at 3 months, 5% at 6 months, 6% at 12 months and 7% at 24 months. Regarding the changes in IOP from baseline, the cumulative probability of having an IOP change ≥10 mmHg was 2.4% at 1–2 weeks, 9.8% at 6–8 weeks, 14.4% at 3 months, 16.6% at 6 months, 21.6% at 12 months, and 28.7% at 24 months **(Fig 2)**.

### Probability of elevated intraocular pressure with repeat injections

**Fig 3:**
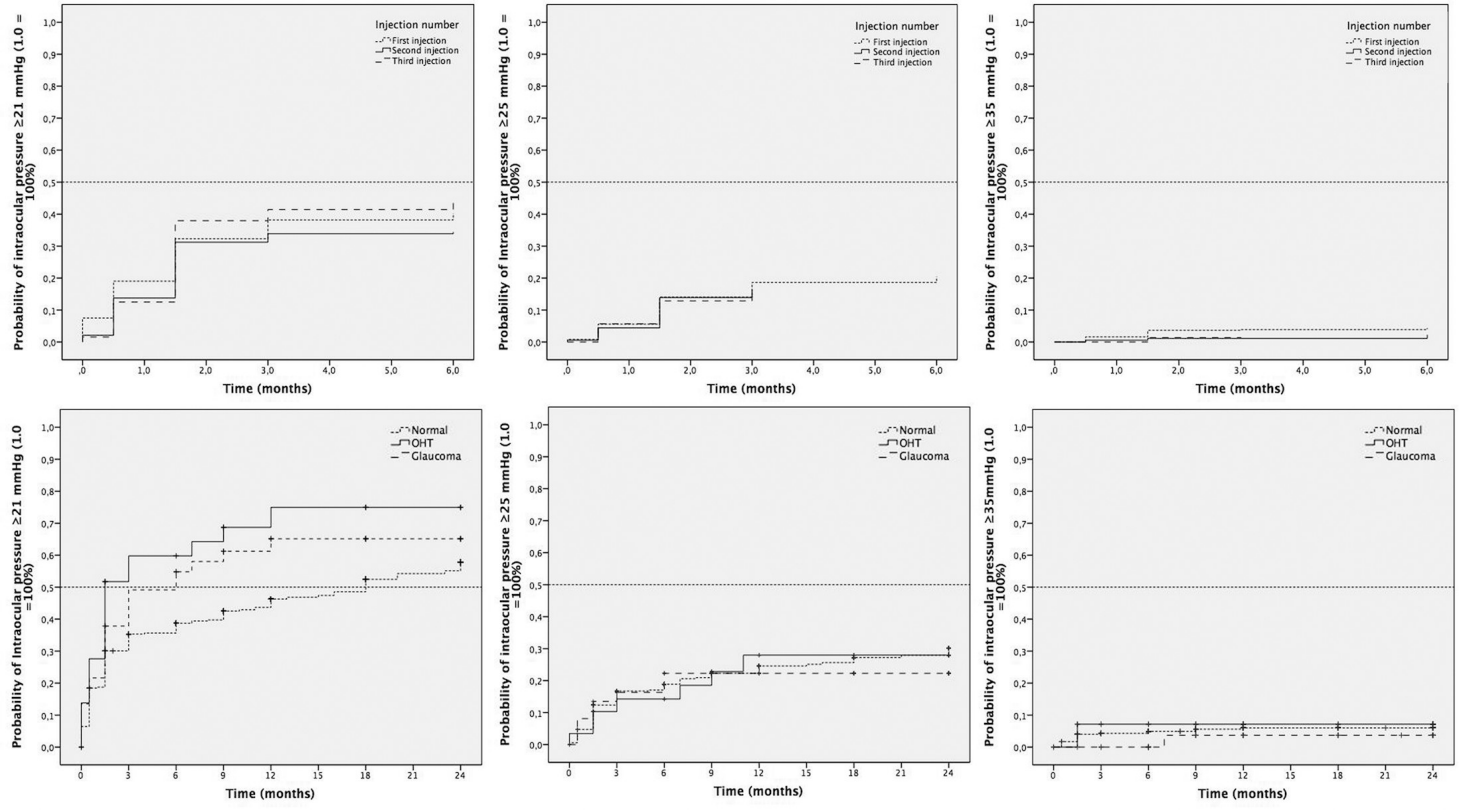
Effect of repeat injections on the changes in intraocular pressure (IOP) in normal, OHT and glaucoma eyes. Top: Kaplan-Meier survival analysis of the time to an elevated IOP and of the change in IOP within 24 months of injection (in months) with the first, second and third injection (dotted line = first injection, solid line = second injection, stripped line = third injection). No differences were observed between any of the injections. Bottom: Kaplan-Meier survival analysis of the time to an elevated intraocular pressure (IOP) and change in IOP within 24 months of injection (in months) in normal, OHT and glaucoma eyes (dotted line = normal eyes, solid line = ocular hypertension eyes, stripped line = glaucoma eyes). Left: Cumulative probability of an IOP ≥21 mmHg, Middle: Cumulative probability of an IOP ≥25 mmHg, Right: Cumulative probability of an IOP ≥35 mmHg. Significant differences were observed at 24 months between normal eyes, and OHT (p = 0.02) and glaucoma eyes (p = 0.01) for IOP ≥21 mmHg (left) but not for ≥25 mmHg (middle, p = 0.91) or ≥35 mmHg (right, p = 0.71). No differences were observed between OHT and glaucoma eyes for any of the IOP levels and time points.

In the overall cohort, a single injection was performed in 58.9% of eyes (253/429), while 25.1% required 2 injections (108/429), 11.4% required 3 injections (50/429) and 4.1% required ≥4 injections (18/429). To evaluate the potential risk of IOP elevations with repeat injections, the cumulative probability of IOP elevation was estimated separately for first, second and third injections. For mild IOP elevation (IOP≥21 mmHg), at 6–8 weeks the probability of this was 32.3% (n = 137), 31.2% (n = 59) and 38% (n = 24) and at 6 months was 40.2% (n = 162), 34.4% (n = 65) and 44.7% (n = 27), respectively **(Fig 3, Top).** The cumulative probability of moderate IOP elevation (IOP ≥25 mmHg) was 14.1% (n = 53), 13.9% (n = 25) and 12.9% (n = 9) at 6–8 weeks and 20.5% (n = 77), 16.4% (n = 29) and 14.3% (n = 10) at 6 months for first, second and third injection. Finally, the cumulative probability of high IOP elevation (IOP ≥35 mmHg) was 3.7% (n = 16), 1.1% (n = 2) and 1.4% (n = 1) at 6–8 weeks, and 4.3% (n = 19), 2.3% (n = 3) and 1.4% (n = 1) at 6 months for the first, second and third injection of the implant, respectively.

### Probability of initiating topical IOP lowering medication and glaucoma surgery

**Fig 4:**
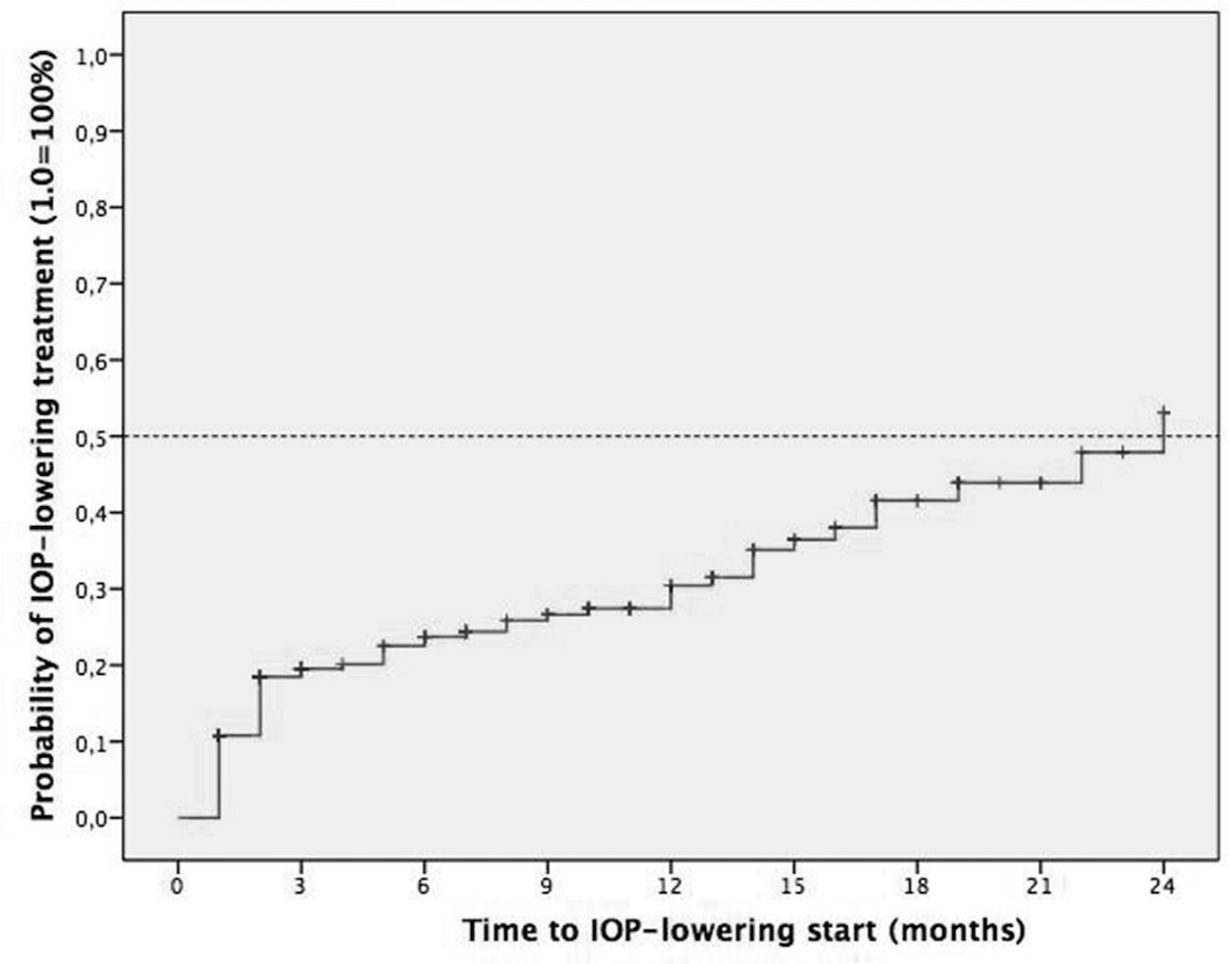
Kaplan-Meier survival analysis of the time to receive IOP lowering medication initiation. Cumulative probability of initiating IOP lowering medication after the first IDI injection.

At baseline, 16.9% of study eyes were already on IOP-lowering medications (72/425). In study eyes that were not on treatment at baseline (n = 353), the cumulative probability of initiating IOP lowering medication was 11% at 1–2 weeks, 19% at 6–8 weeks, 20% at 3 months, 24% at 6 months, 31% at 12 months, 44% at 18 months and 54% at 24 months **(Fig 4)**. The percentage of eyes on IOP lowering medication during the follow-up was 16.9% at baseline (72/425), 19.3% at 1–2 weeks (80/414), 26.4% at 6–8 weeks (109/412), 22.7% at 3 months (88/387), 21.6% at 6 months (83/384), 21% at 12 months (74/351), 19.7% at 18 months (64/324) and 19.6% at 24 months (57/290). Glaucoma surgery was performed in 0.9% (4/429) of the treated eyes, and the techniques employed were non-penetrating deep sclerectomy in 2 cases (50%) and introduction of an Ahmed´s valve in 2 cases (50%). Of these 4 cases, at baseline 2 previously had glaucoma, 1 had OHT and 1 was normal. The indications for treatment in these cases were uveitis, RVO, DME and off-label use (ME after pars plana vitrectomy with epiretinal membrane peel).

### Probability of elevated intraocular pressure in ocular hypertension and glaucoma eyes treated with the dexamethasone implant

The proportion of study eyes which had an IOP ≥21 mmHg at baseline was 6.4% (23/359), 13.8% (4/29) and 13.5% (5/37) for the control, OHT and glaucoma eyes, respectively. After the first IDI injection, the cumulative probability of having an elevated IOP (≥21 mmHg) was 18.5% (n = 66), 27.6% (n = 8) and 21.6% (n = 8) at 1–2 weeks, 30.1% (n = 107), 51% (n = 15) and 37.8% (n = 14) at 6–8 weeks, and 35.3% (n = 125), 59.8% (n = 17) and 49.1% (n = 18) at 3 months, respectively. In the longer term, this cumulative probability was 46.3% (n = 157), 75% (n = 20) and 65.1% (n = 23) at 12 months, and 57.8% (n = 174), 75% (n = 20) and 65.1% (n = 23) at 24 months, differences that were significant (p = 0.01). The cumulative probability of an IOP ≥25 mmHg was 16.7% (n = 59), 14.2% (n = 4), and 16.3% (n = 6) at 3 months, 24.6% (n = 82), 28% (n = 7) and 22.3% (n = 8) at 12 months, and 30.2% (n = 91), 28% (n = 7) and 22.3% (n = 8) at 24 months for normal, OHT and glaucoma eyes (p = 0.91). The cumulative probability of an IOP ≥35 mmHg was 4.3% (n = 15), 7.1% (n = 2) and 0% (n = 0) at 3 months, and 6.0% (n = 20), 7.1% (n = 2) and 3.7% (n = 1) at 12 and 24 months (p = 0.71) (**Fig 3, Bottom).**

## Discussion

This study provides data about the long-term cumulative probability of IOP elevations with the IDI in the real world, and it reveals significant differences in the baseline characteristics of eyes treated in routine clinical care compared to eyes included in the clinical trials. It also highlights the frequency and magnitude of the different levels of IOP elevation, giving an estimate of the cumulative probability of IOP elevation after the first IDI injection, the cumulative risk of IOP elevation with repeat injections and the need for IOP lowering medication and glaucoma surgery in a clinical setting. For the first time, the long-term cumulative probability of IOP elevation in glaucoma and OHT eyes treated with the IDI in routine clinical care are reported.

In our study cohort, significant differences in the baseline characteristics were observed compared to clinical trials (Table 3). In the MEAD study, glaucoma or OHT eyes (defined as IOP ≥23 mmHg without drops or IOP ≥23 mmHg with 1 hypotensive drop) were excluded from the trial. However, 15.6% (67/428) of the eyes already had these conditions in our series and 16.9% were already on IOP lowering medications (72/425) at baseline.[1] In another real world study, the SHASTA study, 31.5% of glaucoma or OHT (91/289) eyes were reported, as well as a 24.2% of eyes on IOP lowering medications (70/289) at baseline. Similarly, the CHROME study included 21.7% of eyes on IOP lowering medication prior to start treatment with the implant.[11,15] Altogether these studies suggest that in the real world the use of the implant is common in eyes where its safety has not been evaluated in the trials, highlighting the need to estimate IOP related problems in routine clinical care, especially in the long-term. Interestingly, the overall mean baseline IOP of the study cohort was similar to the baseline IOP reported in the MEAD trial (15.7±3.4 vs 15.3±2.6 mmHg, respectively) even if a subset of eyes had an IOP ≥ 21 mmHg at baseline (7.9%, 32/402). This suggests that in the series analyzed here the IOP in the glaucoma/OHT eyes was adequately controlled prior to the first IDI injection.

**Table 3:** Intraocular pressure outcomes in clinical trials of dexamethasone implant and in real world studies.

Trial/Study	Indication	Duration (months)	N	IOP≥25 mmHg	IOP≥35 mmHg	IOPchange ≥10mmHg	IOP-lowering medication	Glaucoma surgery
GENEVA	RVO	12	421	-	-	32.8%	35.8%	1.4%
HURON	Uveitis	6	77	10%	5%	-	23%	0%
CHAMPLAIN	DME-PPV	6	55	9%	2%	-	17%	0%
MEAD	DME	36	351	32%	6.6%	27.7%	41.5%	0.6%
BEVORDEX	DME	24	88	-	-	-	22%	0%
CHROME	DME	6*	34	26.5%	2.9%	20.6%	29.4%	1.7%
	RVO	6*	30	26.7%	6.7%	24.1%	16.7%	
	Uveitis	6*	23	17.4%	4.3%	22.7%	8.7%	
SHASTA	RVO	6	289	33.7%	9.4%	32.6%	48.1%**	3.1%***
SAFODEX	All	16.8 ^A^	421	20%	6%	27%	31%	0.7%
	DME	15.7 ^A^	128	11%	3%	16.4%	22.7%	
	RVO	17 ^A^	142	24%	6.3%	33%	38%	
	Uveitis	21.5 ^A^	58	26%	8.6%	38%	34.5%	
	Off-label	16.2 ^A^	20	22%	4%	24.7%	30%	
**This study**	**All**	**24**	**429**	30%*	7%*	**32.8%**	54%*	**0.9%**

*26 weeks

**29.1% related to the implant, according to study investigators

*** 3.1% = 1.4% laser surgery, 1.7% glaucoma incisional surgery

^A^ mean follow up.

We observed the maximum percentage of eyes with an elevated IOP (22.9% of study eyes with ≥21 mmHg) when the dexamethasone release peaked 6–8 weeks after the first injection. At this time point, the percentage of eyes with moderate (≥25 mmHg) and severe (≥35 mmHg) IOP elevations was 9.2% and 2.5%, suggesting that the majority of cases of IOP elevation were mild and well controlled with topical drops at the 1–2 week time point when necessary. It should also be noted that 7.9% of the study eyes (n = 32) already had a baseline IOP ≥21 mmHg, suggesting that these eyes required treatment with the implant due to a partial response or the lack of effect of alternative therapies (i.e. anti-VEGF drugs), which unfortunately is a relatively common situation in the real world. However, the 24-month cumulative probability of having a moderate IOP elevation (≥25 mmHg) or a moderate change in IOP (≥10 mmHg) from the baseline at least once at any time point was 30% and 32.8%, respectively. These figures are highly consistent with data reported in the MEAD trial (32% and 27%), which is the longest follow up and largest IDI clinical trial to date (36 months, n = 351). The data is also in agreement with that form the SHASTA (33.7% and 32.6%) and the SAFODEX study (20% and 27%), the two largest real world studies published to date (n = 289 and n = 421).[1,15,19] Taking into consideration the significant differences between the study cohorts and the lack of exclusion criteria in our series, the data presented appears to be a reliable estimate of the long-term cumulative probability of IOP elevation in the real world.

We did not find a cumulative effect of repeat injections on the IOP of treated eyes. In the SAFODEX study, the percentage of patients with IOP ≥25 mmHg and repeat injections declined over the follow-up, from 5.6% at 2 months to 2% at 24 months, increasing again thereafter to 4.3% at 36 months. This data is consistent with our results, since we describe a cumulative probability and not an overall prevalence at a given time point, which could explain the variability in their study cohort (a lower percentage described at 24 months than at 2 and 36 months). We specifically analyzed the cumulative probability of IOP with each individual injection (first, second and third injection), which revealed that this was no higher with repeat IDI injections. This is an important finding for two main reasons. First, in a real life cohort it is worthwhile considering the possibility of treatment discontinuation if a high IOP response has been obtained with the first implant, which may represent a potential bias and a possible underestimation of this risk with repeat injections. Second, it is not exceptional to find eyes in which all other therapies have failed and that are treated with the IDI implant despite a previous hypertensive response, especially if it was mild and well-controlled with topical drops, as seen in this cohort. Again, this second consideration highlights the importance of gathering real world data to compare such outcomes with those from clinical trials, when eyes with an elevated IOP as an adverse effect are never re-treated with the implant.

In our series, 52% of the study eyes required IOP lowering medication at least once in the 24 months after the first injection. This figure is higher than the 41.5% reported in the MEAD trial, and it may be largely explained by differences in the study cohorts, in particular to the exclusion criteria applied in the clinical trial.[1] Interestingly, the SHASTA study observed a similar rate (48.1%) with a shorter follow-up (6 months), suggesting that this rate does not appear to vary considerably in the long-term, as indicated in the MEAD trial where no cumulative response in terms of IOP elevation was observed with repeat injections.[15] The rate of eyes that required glaucoma surgery was very low (0.9%) and while consistent with the MEAD data (0.6%), it is lower than the figures reported in the SHASTA (3.1%) and CHROME (1.4%) studies.[1,11,15] This could be explained by the higher percentage of glaucoma and OHT eyes included at baseline in these earlier study cohorts (e.g. 31.5%, 91/289 in SHASTA). In our cohort, we did not observe any association with treatment indication, and 3 of the 4 eyes that required surgery had predisposing conditions (2 glaucoma and 1 OHT).

One of the main findings of this study is that it provides long-term estimates of how IOP behaves in glaucoma and/or OHT eyes treated with the IDI implant. Although these conditions have traditionally been considered exclusion criteria in clinical trials, the vast majority of real life studies include a subset of glaucoma and/or OHT eyes that have received treatment with the implant in routine clinical care.[9,11,15] There is a paucity of data in the literature about the IOP outcomes in this specific subgroup, as only few studies on small cohorts of patients have been reported, and none of them have provided meaningful long-term data.[20,21] Interestingly, we found a higher cumulative probability of mild IOP elevation (≥21 mmHg) in OHT and glaucoma eyes compared to normal eyes at 12 (75% vs 65.1% vs 46.3%) and 24 months (75% vs 65.1% vs 57.8%). However, no differences were observed for moderate (≥25 mmHg) or high IOP elevations (≥35 mmHg) at any of these time points (28% vs 22.3% vs 24.6% at 12 months and 28% vs 22.3% vs 30.2% at 24 months, and 7.1% vs 3.7% vs 5.6% at both time points, respectively). Since the proportion of eyes with an elevated IOP level (≥21 mmHg) at baseline was already higher for OHT (13.8%) and glaucoma eyes (13.5%) relative to the normal eyes (6.4%), these data highlight that the magnitude of the IOP spike does not appear to be significantly different in OHT or glaucoma eyes compared to normal eyes, especially in terms of moderate or high IOP levels. This previously unreported long-term data is important for patient counselling, as the potential risk for untreatable glaucoma is a significant concern in these eyes, when other therapies have often failed to control the disease (i.e. in refractory DME or severe uveitis).

As with other real life series, this study has a number of limitations. First, data collection was retrospective with a significant risk of missing some data not collected at some visits. Second, the variable follow-up may have influenced the time to repeat IDI injections and therefore, its potential side effects, namely elevations in IOP that could therefore be underestimated. Third, the cases were collected from two different specialist units with different clinician guided indications for treatment and re-treatment criteria. Fourth, a special selection bias may have occurred since the cases included in this study were collected in tertiary referral units and they may not reflect those seen in a standard general ophthalmology practice, a limitation referred to as Berkson´s bias. Finally, potential confounding factors such as concomitant pathologies were not specifically excluded (as in clinical trial settings), as data was collected in a routine clinical care setting.

In summary, this study provides real world data about the long-term cumulative probability of IOP elevation in a large cohort of eyes treated with the IDI in routine clinical care. The frequency and magnitude of IOP elevation reported appears consistent with the data described in the largest clinical trial, albeit with notable differences between the study cohorts. Whereas the need for IOP lowering medication appears greater in the real world, the rate of glaucoma surgery was very low and again, consistent with clinical trials. Repeat injections of the IDI do not appear to increase the risk of IOP elevation. For the first time, we present the long-term cumulative probability of IOP elevation in glaucoma and OHT eyes treated with the IDI in routine clinical care, which appears higher for mild but not for moderate and severe elevations in IOP. This study highlights the need for real world data to evaluate the profile of adverse effects related to drug use beyond the scenario of clinical trials and in a routine clinical care setting.
